# SPECTRA: A Novel Compact System for Surface Plasmon Resonance Measurements

**DOI:** 10.3390/s23094309

**Published:** 2023-04-26

**Authors:** Elisabetta Pasqualotto, Erica Cretaio, Lara Franchin, Alessandro De Toni, Alessandro Paccagnella, Stefano Bonaldo, Matteo Scaramuzza

**Affiliations:** 1 ARC—Centro Ricerche Applicate s.r.l., 35132 Padova, Italy; 2 Department of Information Engineering, University of Padova, 35131 Padova, Italy; 3 Up-Code s.r.l., 35132 Padova, Italy

**Keywords:** surface plasmon resonance, optical sensing, compact system, gratings, SPECTRA

## Abstract

Surface plasmon resonance (SPR) is a common and useful measurement technique to perform fast and sensitive optical detection. SPR instrumentations usually comprise optical systems of mirrors and lenses which are quite expensive and impractical for point-of-care applications. In this work, we presented a novel and compact SPR device called SPECTRA, designed as a spectrophotometer add-on with a grating coupling configuration. The device is conceived as a marketable solution to perform quick SPR measurements in grating configuration without the requirement of complex instrumentation. The device can be customized either in a vertical structure to reach lower incident light angles, or in a horizontal configuration, which is suitable for SPR analysis using liquid solutions. The SPECTRA performance was evaluated through SPR measurements in typical applications. The vertical SPECTRA system was employed to detect different functionalization molecules on gold 720 nm-period grating devices. Meanwhile, the horizontal SPECTRA configuration was exploited to carry out fluid-dynamic measurements using a microfluidic cell with glycerol solutions at increasing concentrations to account for different refractive indexes. The experimental tests confirmed that the SPECTRA design is suitable for SPR measurements, demonstrating its capability to detect the presence of analytes and changes in surface properties both in static and dynamic set-ups.

## 1. Introduction

Surface plasmon resonance (SPR) is a valuable optical analytic tool used as a sensing technique in a wide range of applications including studies of biomolecular interactions [1,2], drug discovery [3,4], and pathogen detection [5,6]. In recent years, there has been an ever-growing interest in optical detection techniques such as absorbance [7,8,9] and SPR [10,11,12], since they guarantee high sensitivity, immunity to external disturbance, stability, and low noise [13]. The SPR measurements allow rapid, sensitive, real-time, and label-free analysis, and they can also be applied in an array to detect analytes simultaneously [14]. SPR measurements are typically carried out by using two configurations: the prism-coupled SPR, also called Kretschmann configuration [15], and the grating-coupled configuration [16,17,18,19,20]. In the Kretschmann configuration, a high refractive index prism is exploited in order to increase the incident light momentum and achieve the SPP-momentum matching [16]. Several oils characterized by different dielectric constants and refractive indexes can be employed at the prism/metal interface to ensure the SPP-momentum matching with more complex and expensive set-ups [16]. Meanwhile, the grating-coupled configuration uses a nanostructured grating sensor for the generation of SPPs in a metal/dielectric interface [16,17,18,19,20,21]. The SPR occurs when the on-plane momentum component of a particular diffracted order matches the SPP momentum, and it depends on the refractive index of the dielectric in contact with the metal surface [19,20,21]. The SPR analysis must be performed in a specialized laboratory using a well-calibrated custom-made system of mirrors and lenses or a commercially available device, which are usually expensive. Hence, more compact SPR approaches need to be implemented to satisfy the increasing requirements of portability and rapidness for the detection systems. To our knowledge, in the literature, only few works reported portable and compact SPR devices [20,21,22,23,24,25,26]. E.g., Ref. [24] describes a portable SPR system for the real-time monitoring of a wide range of biological analytes, which is 4 cm long and comprises a Kreschmann configuration. However, these devices are mainly developed as part of a task-specific detection system, and they are not conceived to be measurement instruments.

In this work, we propose the SPECTRA, which is a novel and compact add-on device for a common spectrophotometer capable of performing quick SPR analysis with a grating coupling configuration. The device is available either in a horizontal or a vertical structure, and it is designed as a spectrophotometer add-on, exploiting the instrument as light source and detector. The system’s main features and applications are explored through experimental measurements, proving the merit of this novel and economic SPR instrument.

## 2. The Proposed System

**Figure 1:**
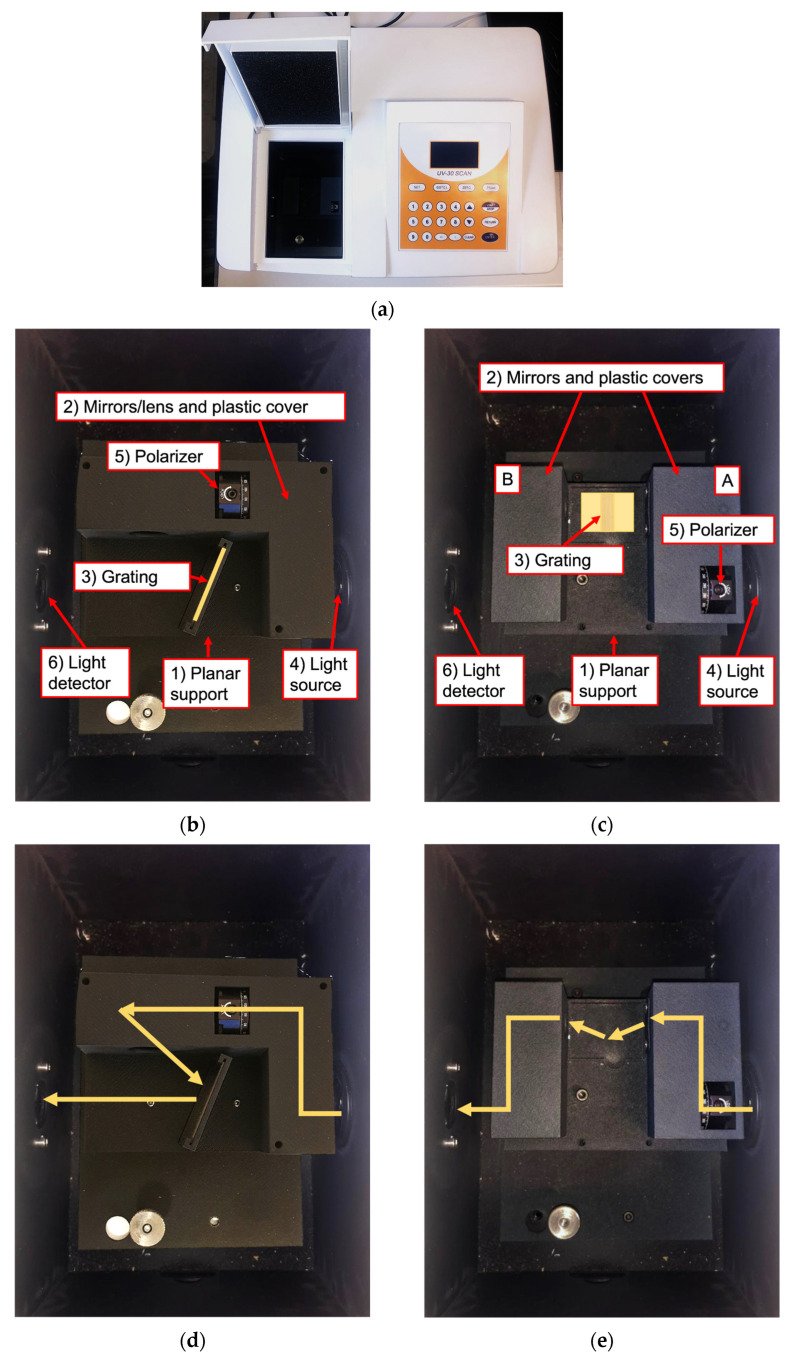
The SPECTRA device. (**a**) The SPECTRA inside the spectrophotometer; (**b**) The vertical SPECTRA structure; (**c**) The horizontal SPECTRA structure; (**d**) and (**e**) Respectively, the vertical and horizontal SPECTRA light beam paths.

The SPECTRA system is a novel portable optical bench specifically designed to carry out easy and quick SPR measurements as a spectrophotometer add-on, as shown in Figure 1a. Its dimensions are 102 mm of width, 136 mm of depth, and 75 mm of height. The system allows to perform SPR measurements using the grating configuration, avoiding the traditional complex and bulky SPR set-ups. Through the device, the SPR measurements are carried out as a function of incident light wavelength, fixing the light polarization at a given incident angle. The SPECTRA can be applied easily to analyze a sensing device interface, studying the different chemical-physical phenomena on the surfaces under examination such as the presence of nanostructures and molecular adsorbance on metal and polymers. The SPR measurements must be carried out through the spectrophotometer, and the proposed device is exploited to focus the light beam on the surface being tested and generate the surface plasmons. Hence, the sensitivity of the measurements is related to the spectrophotometer only.

The device is available in two different structures. The vertical structure requires a lower incident light angle (~20°), thus generating a deeper plasmonic dip. Meanwhile, the horizontal configuration is more suitable to perform measurements in the presence of solutions, even allowing dynamic set-ups with microfluidic cells. The vertical optical device (Figure 1b) comprises a rectangular planar support (1), three planar mirrors (2), and a bi-concave lens (3). The grating (4) must be positioned vertically in the special support at the center of the plane. The light beam is projected on the first mirror by the spectrophotometer source (5) through a polarizer (6). Mirrors and lens are protected by a plastic cover to ease the device handling. The mirrors are positioned in an L shape in order to focalize the light beam firstly on the tested device surface and then on the spectrophotometer detector (7). The horizontal optical system (Figure 1c) is similarly composed of a rectangular planar support (1) and four mirrors (2). In this case, the grating under test (3) must be positioned horizontally on the center of the plane into an indent to maximize the incident light. Two mirrors are positioned on the right side (A) in order to focalize the polarized light beam on the test device surface, while the other two mirrors are located on the left (B) to orient the reflective light on the detector (6). For further clarification of the add-on functionality, we highlighted the light beam path in both configurations in Figure 1d,e.

## 3. Experimental Tests

### 3.1. Vertical Configuration

**Figure 2:**
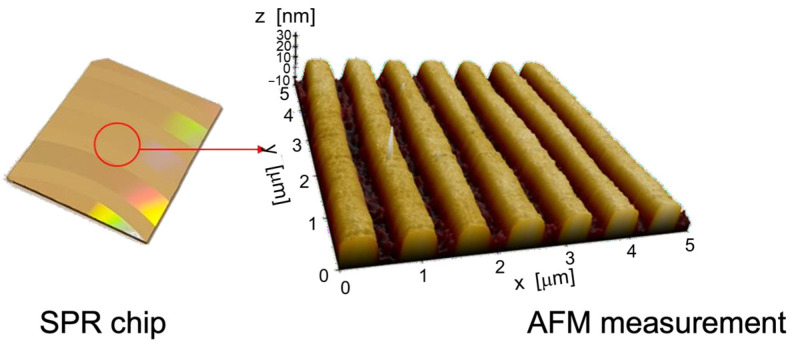
The gold gratings used for the SPR measurements. The figure on the right side shows the sensor profile measured by AFM, which evidences the 720 nm period of the nanostructured sensor.

The vertical SPECTRA configuration allows to characterize surfaces at a low resonance angle. In order to assess the proposed device influence on SPR measurements, we carried out experimental tests using custom-made gold grating sensors. The grating sensors were fabricated by sputtering 50 nm gold over a nanostructured polycarbonate substrate having a 720 nm period, as shown in Figure 2. Their production was based on polycarbonate of optical disks, which were customized to have a continuous grating with specific period covered by a gold layer, which ensures great affinity with biomolecules.

The measurements were carried out with the SPECTRA system installed into a spectrophotometer from Mapada Instruments, model UV-1600. The spectrophotometer is provided with a tungsten source light with capabilities of measuring in the wavelength spectra ranging from 320 nm to 1100 nm. The SPR signals of bare gratings were firstly characterized, then their surface was treated and functionalized with different antibodies to evaluate the system’s capability to detect the plasmonic peak variation.

#### 3.1.1. System Validation

**Figure 3:**
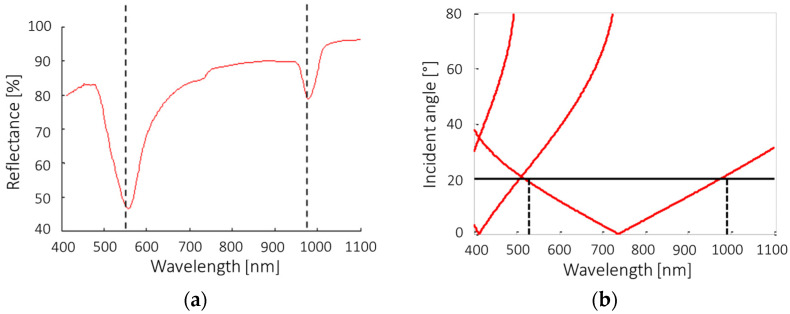
(**a**) Air reflectance of 720 nm period gold gratings; (**b**) Vectorial model of the plasmonic peaks as a function of incident light angle (θ_i_) and wavelength. The black dashed lines show the wavelength of the peaks.

The system measurement capability was evaluated through comparison with the vectorial model of the plasmonic resonance. The position of the plasmonic peaks in air was measured for gold gratings with a period of 720 nm using SPECTRA as a spectrophotometer add-on. The experimental measurements were repeated three times, then the plasmonic peaks position was extrapolated. The measurement of a flat gold surface has been considered as baseline for the spectrophotometer. Results are compared with simulated data obtained through a vector model of SPR dispersion law [19,20]:

(1) $$k_{SPPs}=\frac{2\pi }{\lambda }\sqrt{\frac{\varepsilon_{d}\varepsilon_{m}}{\varepsilon_{d}+\varepsilon_{m}}}$$

where *k_SPPs_* is the SPP momentum, *λ* is the wavelength of the incident light, *ε_d_* and *ε_m_* are the complex dielectric constants of the material over the sensor and of the metal (gold), respectively. Figure 3a shows that the experimental peaks occur at 520 nm and 990 nm of wavelength, which is perfectly correspondent to the positions identified through the vectorial model (Figure 3b).

#### 3.1.2. Sensor Biofunctionalization

**Figure 4:**
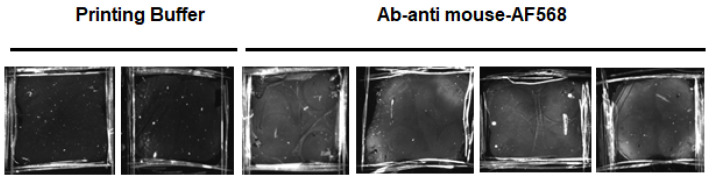
The sensor surface functionalized with the anti-mouse-AF568 antibody or without antibody (printing buffer). In the presence of antibody after the excess removal, it is possible to observe a fluorescence signal caused by the immobilization of the fluorescent probe on sensor surface.

The SPR sensors were functionalized according to the following procedure. Each gold surface was cleaned by immersion in base piranha solution (5:1:1 mixture of H_2_O, NH_4_OH, H_2_O_2_) for 15 min [27,28]. The sensors were incubated overnight with the MUA spacer (MUA 2.5 mM in Absolute EtOH). After the removal of spacer excess, the SAM carboxyl groups were activated with EDC and s-NHS (5 mM solution in MES buffer, 100 mM MES, 500 mM NaCl pH6.0) for 15 min. The surfaces were then rinsed and incubated with the anti-mouse-AF568 antibody diluted in printing buffer (final concentration 100 mM Na3PO4, 300 mM NaCl, 0.01% Triton X-100 pH 7.2). The antibody excess was removed, and the sensor surfaces were passivated with blocking buffer (50 mM sodium phosphate, 1 M Ethanolamine, pH 7.2) for 1 h at RT. The presence of antibodies on the surface was verified through a LED scanner to detect the fluorescence signal associated with the Alexa Fluorophore 568. All sensor surfaces were scanned for an acquisition time of 100 milliseconds (Figure 4). The functionalization procedure was also followed step by step in SPR as described below.

#### 3.1.3. Influence of Surface Functionalization

**Figure 5:**
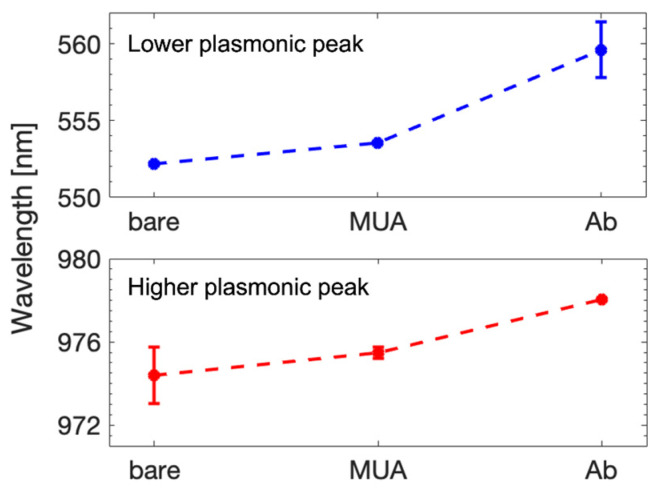
Variation of plasmonic peak position at each functionalization step. The top plot shows the lower peak shift, and the bottom plot shows the higher peak shift.

The bare 720 nm gold gratings were firstly measured in air through the spectrophotometer using SPECTRA as add-on. As expected from the results in Section 3.1.1, two different plasmonic peaks were identified at 552.19 nm and 974.38 nm of wavelength. The gratings’ surface was then incubated with 11-Mercaptoundecanoic acid (MUA) for 8 h. Finally, the surface was functionalized with anti-mouse-AF568 antibody, according to the procedure previously described. The same experiment was repeated at each step and the results are reported in Figure 5 as mean and standard deviation. It can be easily seen that both peaks are influenced by the subsequent functionalization. The position shift of the lower peak is about 1.36 nm for the MUA and reaches 7.4 nm in the presence of antibody. The higher peak wavelength changes by some nm as well. These results show that the vertical system is able to detect variation of the gratings’ surface due to molecular adsorbance and biological elements.

### 3.2. Horizontal Configuration

**Figure 6:**
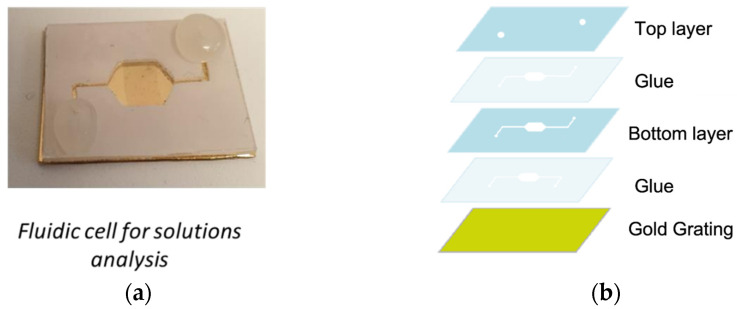
(**a**) The microfluidic cell used for SPR measurements with aqueous solutions. (**b**) The structure of the microfluidic cell.

The horizontal SPECTRA configuration results are more suitable for SPR measurements in solution due to the planar structure. Hence, we carried out experimental tests in order to demonstrate the device’s operation in the presence of aqueous solutions. The measurements were performed using a custom-made microfluidic cell (Figure 6) integrating the 720 nm gold gratings. The microfluidic cell was made of two polycarbonate layers (thickness 0.6 mm). The bottom layer was cut to obtain the fluidic channels and the cell, while two holes were cut on the top layer edges to allow the fluid injection. The final polycarbonate structure was glued to the grating. A system of tubes, syringe, and a peristaltic pump (VELP SCIENTIFICA SP311) was connected to the microfluidic cell through the holes.

Firstly, we performed SPR tests varying the refractive index of the solution to assess the influence of this parameter on the measurements. Then, we let the solution flow into the microfluidic cell evaluating the influence of a dynamic set-up on the SPECTRA SPR measurements.

#### 3.2.1. Influence of Refractive Index

Since the SPECTRA horizontal configuration allows simple SPR analysis in the presence of solutions, we carried out test solutions of ultrapure water (de-ionized and filtered at 0.22 μm water with conductivity < 2000 μS/cm) and glycerol in different percentages (glycerol percentages: 0%, 5%, 10%, 15%, 30%), evaluating the SPECTRA system capability to detect refractive index variations in a range of wavelengths between 400 nm and 100 nm. The refractive index (n) of the different solutions is reported in Table 1.

**Figure 7:**
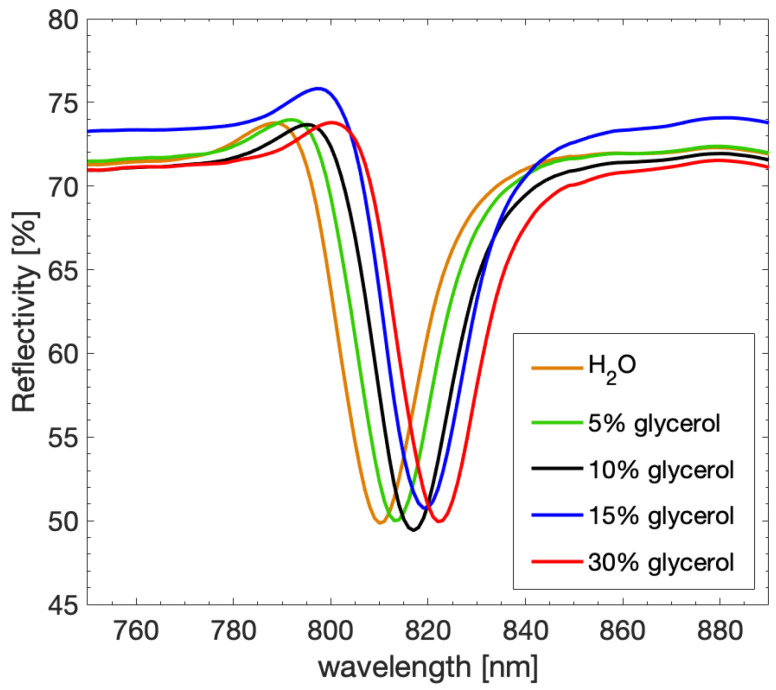
Reflectivity as a function of wavelength for aqueous solutions at increasing glycerol concentration with a grating period of 720 nm.

The reflectivity for different glycerol solutions obtained through the SPECTRA system is presented as a function of wavelength (Figure 7). All the peaks show similar height (~22%) and amplitude (~17 nm), and the increase in refractive index can be followed from the peak shift towards higher wavelengths. The water solution presents the minimum reflectance at 810 nm, while the glycerol solutions evidence shifts in the peak position, reaching 822 nm for 30% glycerol concentration.

#### 3.2.2. Influence of Dynamic Measurements

**Figure 8:**
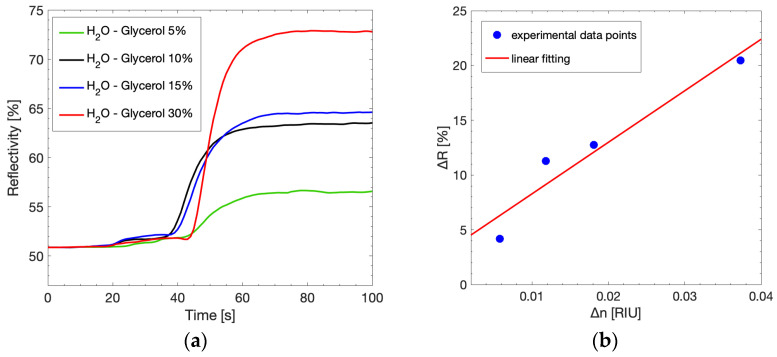
(**a**) Reflectivity acquired at λ = 808 nm as a function of time in the dynamic set-up. The water solution (H_2_O) is substituted by solutions at increasing glycerol concentration, thus leading to a change in the reflectivity; (**b**) ∆*R* as a function of ∆*n* during the dynamic measurements. The red line shows system sensitivity.

In order to assess the system’s capability to detect sudden changes in the solution refractive index, we tested the system with the microfluidic cell using glycerol solutions at different concentrations. The solutions were injected through a peristaltic pump connected to the microfluidic cell. The acquisition of the plasmonic peaks was performed over an observation time of 100 s (Figure 8a). The light wavelength was fixed at 808 nm, which corresponds to the plasmonic peak of the 720 nm grating, and the reflected light was acquired. Firstly, a water solution was injected into the microfluidic cell. Then, after 30 s, the peristaltic pump started to inject the glycerol solution.

The same experiment was performed for four increasing glycerol concentrations (5%, 10%, 15%, 30%). For all the samples, the reflectivity spiked rapidly, reaching the maximum value around 60 s and remaining constant overall until the end of the observation time. The reflectivity values, the respective variation of reflectivity ∆*R*, and the variation of reflective index between water and glycerol solutions are calculated and reported in Table 2. As expected, all the samples show an increase in ∆*R* and ∆*n* proportional to the glycerol concentrations. The correlation between ∆*R* and ∆*n* can be seen in Figure 8b, evidencing a linear behavior. Hence, the system sensitivity can be estimated from the linear pendency, and it is calculated as 470.5%/RIU.

## 4. Conclusions

This work aimed to present the main features of SPECTRA, which is a novel and compact system to perform SPR measurements. Our device was conceived as a spectrophotometer add-on, avoiding complex traditional SPR set-ups. The SPECTRA allows to perform SPR analysis using a grating configuration in a range of wavelengths, with a fixed polarization angle. The device is available as a vertical or horizontal structure, which present different characteristics. The vertical system allows to reach lower incident light angles, while the horizontal system is suitable for SPR analysis in solution. Experimental tests on the vertical device confirmed the system capability of detecting variations of plasmonic peak due to surface functionalization. Meanwhile, the horizontal configuration highlighted the system capability of detecting variations of plasmonic peak due to the reflective index in both static and dynamic set-ups. The sensitivity of the system is related to the spectrophotometer detector. Hence, the proposed device proved to be a useful and reliable tool to perform SPR measurements on grating surfaces in a compact and quick way. Future work will be focused on SPECTRA adoption for sensing applications and the development of different SPECTRA structures to allow SPR analysis with prismatic configurations such as Kreschmann.

## 5. Patents

SPECTRA is patented EP3462163A1; IT201700109902A1. Assignee is Up-Code s.r.l.

## Figures and Tables

**Table 1:** Extrapolated reflectivity parameters for each solution variation. The reflective index is retrieved from the measurements.

Glycerol Concentration	0%	5%	10%	15%	30%
**n [RIU]**	1.3333	1.3388	1.3448	1.3511	1.3703

**Table 2:** Extrapolated reflectivity parameters for each solution variation. The variation of reflectivity and of the reflective index are retrieved from the measurements.

Solution	Min *R* [%]	Max *R* [%]	∆*R* [%]	∆*n* [RIU]
H_2_O to 5% glycerol	50.9	55.07	4.17	0.0058
H_2_O to 10% glycerol	50.92	62.22	11.29	0.0118
H_2_O to 15% glycerol	50.95	63.69	12.74	0.0181
H_2_O to 30% glycerol	50.93	71.40	20.47	0.0373

## Data Availability

Data are not publicly available due to confidentiality reasons.
